# Simulation of dyslexia. How literacy and cognitive skills can help distinguish college students with dyslexia from malingerers

**DOI:** 10.1371/journal.pone.0196903

**Published:** 2018-05-21

**Authors:** Madelon van den Boer, Elise H. de Bree, Peter F. de Jong

**Affiliations:** Research Institute of Child Development and Education, University of Amsterdam, Amsterdam, The Netherlands; University of Windsor, CANADA

## Abstract

Academic accommodations associated with a diagnosis of dyslexia might be incentives for college students without reading or spelling difficulties to feign dyslexia and obtain the diagnosis unfairly. In the current study we examined malingering practices by comparing the performance of college students instructed to malinger dyslexia (*n* = 28) to that of students actually diagnosed with dyslexia (*n* = 16). We also included a control group of students without reading and spelling difficulties (*n* = 28). The test battery included tasks tapping literacy skills as well as underlying cognitive skills associated with literacy outcomes. These tasks are commonly used in diagnosing dyslexia. We examined patterns in the performance of malingerers across tasks and tested whether malingerers could be identified based on their performance on a limited number of tasks. Results indicated that malingerers scored significantly lower than students with dyslexia on reading and spelling skills; i.e., the core characteristics of dyslexia. Especially reading performance was extremely low and not in line with students’ age and level of education. Findings for underlying cognitive skills were mixed. Overall, malingerers scored lower than students with dyslexia on tasks tapping mainly speed, whereas the two groups did not differ on tasks reflecting mainly accuracy. Based on word and pseudoword reading and letter and digit naming, the three groups could be distinguished with reasonable sensitivity and specificity. In all, results indicate that college students seem to understand on which tasks they should feign dyslexia, but tend to exaggerate difficulties on these tasks to the point where diagnosticians should mistrust performance.

## Introduction

For college students with dyslexia, academic accommodations are often available to compensate the effect their reading and spelling difficulties might have on academic learning. These accommodations are necessary for students diagnosed with dyslexia, but also appeal to students who do not experience reading or spelling difficulties. As these benefits might even outweigh the disadvantages of having the diagnosis dyslexia, there is growing concern that students may attempt to feign symptoms of dyslexia to obtain a diagnosis and thereby access to various forms of support. These forms of support are most often provided, at least in the Netherlands, when an official diagnostic report can be presented at the administration office. Although the report is verified, there is no additional testing to verify the actual diagnosis. Therefore, if students succeed in feigning dyslexia during testing, they are able to profit from various forms of support for their entire student careers.

It is not known how often students feign dyslexia and obtain a diagnosis. Sullivan et al. [1] concluded that about 15% of the patients who received the broader diagnosis learning disability had exaggerated their symptoms, although this estimate should be considered cautiously given a small sample size. The growing concern for feigning practices has mainly been fueled by the increase in the number of people diagnosed with dyslexia over the last few years. Such feigning practices are of course of great concern. Not only might certain students obtain academic degrees under unfair circumstances, but more importantly, it would harm the reputation of students who really do have dyslexia. The question is, however, how well dyslexia can be feigned. In this study we examine performance on literacy skills and related cognitive skills by college students instructed to malinger dyslexia to better understand and appraise potential feigning strategies.

Dyslexia is characterized by difficulties with accurate and/or rapid word reading and/or spelling despite adequate instruction, and in the absence of general cognitive or sensory deficits [2,3]. In previous studies it has been found that students are able to feign the core symptoms of dyslexia, especially on the most commonly used literacy tests. They were found to score as low as or lower than students with dyslexia on reading and spelling tests [4–6]. As a result, it is likely that malingerers would be diagnosed with dyslexia, since diagnostic assessment of dyslexia is by and large confirmatory. In other words, diagnostic assessment is mainly focused on accumulating evidence that students show signs of dyslexia, such as scores within the lowest 10% on reading and/or spelling.

Criteria that would rule out dyslexia are less common. One method to provide a contra-indication for dyslexia is the use of a performance validity test. A performance validity test is a task that is not related to dyslexia (i.e., both readers with and without dyslexia perform well on this task), but evokes poor performance in malingerers because they would consider the task to tap skills related to the disorder. As such the performance validity test can clarify whether a person’s test performance is reflective of their actual level of ability. These tests are sometimes also referred to as *symptom* validity tests, but we follow the terminology of Larrabee [7], who suggested to use performance validity to refer to validity of test performance and symptom validity to refer to validity of symptoms as reported on self-report measures.

A few studies have looked into performance validity tests in adult populations. Osmon et al. [8], for example, developed a word reading test that distinguished between students feigning reading difficulties and typical readers. In this test participants were shown a word for three seconds followed by a short delay and were then presented with two alternatives. Participants were asked to indicate which word they had just seen. Distractor items consisted of words containing a mirror image letter, an orthographically similar letter, or an illegal letter combination. Whereas typical readers hardly made any errors, the feigners almost all made a considerable number of errors. Feigners were thus adequately distinguished from typical readers. This study did not include students with an actual diagnosis of dyslexia, precluding a conclusion about the distinction between feigners and students with dyslexia.

Harrison and colleagues [4,5] also applied performance validity testing. They used a passage reading task including normal and scrambled passages. In the latter, letters in longer words were scrambled. On this test feigners read significantly more slowly and made more errors than students with dyslexia. A more accurate distinction between the groups could be achieved with the use of a Feigning Index. This index consists of a list of features more frequently observed in those feigning dyslexia than in those who actually have dyslexia both in the experimental reading task and in standardized reading tasks (e.g., errors in reading tasks, low reading fluency on easy passages, but also poor visual matching and low decision speed). Feigners scored significantly higher on this index than either typical readers or students with dyslexia.

Although performance validity tests seem to distinguish between feigners and both typical readers and students with dyslexia, it is not yet clear which test would be best and whether all feigners could be identified with a single test. Furthermore, these tests are not commonly available. In the current study we therefore examine whether feigners can also be identified based on the symptoms and risk factors commonly assessed in diagnosing dyslexia. Instead of developing a new task that might work as performance validity task, we presented students asked to malinger dyslexia with a test battery that is commonly used to diagnose dyslexia in higher education. We examined whether students malingering dyslexia could convincingly present themselves as a student with dyslexia by looking at their performance across several indicators of dyslexia. The main question is whether malingerers display the same pattern of strengths and difficulties in test performance as students with dyslexia. Regarding reading and spelling, the core symptoms of dyslexia, it has been shown that malingerers tend to perform extremely poorly [4–6]. In the current study we examined whether malingerers can be identified, even when they do not perform overly poorly, based on the pattern of their scores across literacy skills and associated cognitive skills. Credible scores on associated skills might be more difficult to feign, especially since these scores should be in line with scores on the literacy tasks for a diagnosis of dyslexia.

One previous study has taken a similar approach. Lindstrom et al. [6] found that students were quite able to simulate dyslexia on measures commonly used in diagnosing dyslexia. Their scores were indistinguishable from students with dyslexia on measures of word and pseudoword reading accuracy, spelling, and also intelligence. The groups could be distinguished on reading fluency, however, as students simulating dyslexia tended to perform much more poorly than those with dyslexia. Interestingly, malingerers did not show a deficit in phonological awareness, a cognitive factor associated with dyslexia.

In the current study, we aimed to look more closely not only at performance on different tests, but also at the pattern of performance across tasks, especially variants of the same task. Based on previous studies [4–6], we expected malingerers to simulate dyslexia successfully on the literacy tasks, or to exaggerate the symptoms of dyslexia, resulting in extremely poor performance. However, simulating dyslexia similarly on various reading tasks might be more difficult than simulating on a single task. We also assessed several cognitive factors that could underlie the development of the disorder, both those commonly used in dyslexia assessment, and new tests that appear promising for diagnosing dyslexia. Malingerers might have more difficulty simulating dyslexia on these tasks, as these tasks are not as clearly associated with the disorder as literacy skills are.

A deficit in processing phonology is generally considered an important risk factor for dyslexia in both children and adults [9–11]. Two cognitive skills that are seen as indicative of phonological processing skills, and that are generally assessed in diagnosing dyslexia, are phonological awareness and rapid automatized naming (RAN). Phonological awareness, the ability to distinguish and manipulate sounds in spoken words, is associated with reading and spelling performance across ages and languages, especially when both accuracy and speed are taken into account [12,13]. RAN is the ability to quickly name a set of familiar symbols (i.e., letters or digits). It is associated with reading performance across ages and languages [14]. Phonological awareness has received substantial attention as a risk factor for dyslexia [11,15,16]. It might therefore be a familiar task and malingerers could be expected to resemble students with dyslexia on this task. However, given that phonological awareness tasks are oral tasks, focusing only on spoken language, not all malingerers might see the relation with reading and spelling performance (see also [6]). Concerning RAN, malingerers might associate naming of letters with dyslexia, but difficulties in naming digits might be overlooked as indicator of dyslexia. This would result in a specific pattern of performance on the RAN tasks, that is malingerers naming letters more slowly than digits.

A third skill that can be seen as indicative of phonological processing skills is verbal short-term memory [17]. Individuals with dyslexia have been shown to perform worse than typical readers on tasks tapping verbal short-term memory, most often measured as nonword repetition [18]. However, it was also found that this difference is largely explained by the lower oral language skills in part of the individuals with dyslexia, mainly those who were also diagnosed with specific language impairment. In general, it has been found that verbal short-term memory is related to reading abilities, but that this relation is weaker than the relation of reading with other phonological skills, such as phonological awareness and RAN [19,20]. As a result, the hypotheses concerning verbal short-term memory are not so clear. Students with dyslexia, as a group, are expected to score lower on verbal short-term memory, but malingerers are not necessarily expected to associate this task with dyslexia, and thus might not simulate dyslexia on this task.

In addition to these ‘usual suspects’, there are two other cognitive skills that have recently been put forward as potential predictors of reading ability and dyslexia: the Stroop task and the visual attention span. These tasks are not included in typical diagnostic assessment of dyslexia. In the color-word Stroop interference task [21], naming of a color is found to be slowed down by an incongruent printed word (e.g., the word ‘*green’* printed in red ink). It has long been assumed that reading skill is positively related to Stroop interference, such that better readers exhibit stronger interference, whereas beginning readers or poor readers show less interference, because activation of the word is too slow [22]. Recent findings, however, show the opposite pattern; better reading skills are associated with less interference in both children [23,24] and adults [25]. The explanation is that reading is a more practiced skill than naming colors [26]. Therefore, reading the word is the dominant response upon encountering a written word in colored ink. Irrespective of reading ability, the word is processed first, but then has to be inhibited to activate the alternative, correct response, the color name. The more slowly the word is identified, the longer it takes before this response can be inhibited and the alternative response can be activated. In other words, everyone first reads the word and then names the color. For poor readers, reading the word takes more time, resulting in longer latencies for color naming and thus in larger interference effects. We thus expect students with dyslexia to present with larger interference effects than typical readers. We expect malingerers to have difficulty feigning dyslexia on this task and thus to resemble the typical readers, since this task taps rather automatic responses.

In addition to the Stroop tasks that are typically used to establish the interference effect, in this case naming of colors and naming the colors of words in incongruent ink color, we administered an additional Stroop condition not commonly part of the Stroop task. In this condition we presented participants with the words printed in incongruent colors, but this time asked them to read aloud the words. As this task clearly involves reading, we expect malingerers to simulate dyslexia on this task specifically, and thus to perform similarly to or worse than students with dyslexia. This would also mean that when all three versions of the Stroop task are analyzed simultaneously, the pattern of performance is different for the malingerers as compared to the students with dyslexia and the typical readers.

The second cognitive skill that has been related to dyslexia in addition to phonological processing skills is visual processing [27]. Prominent among visual theories is the visual attention span hypothesis [28]. This hypothesis states that the visual attention span (VAS), the number of orthographic units (i.e., letters, letter clusters, syllables) that can be processed simultaneously at a glance, is an important cognitive skill underlying reading performance. A large VAS allows words to be processed in parallel and thus to be identified through sight word reading, whereas a small VAS requires visual attention to be focused on sublexical units, resulting in serial word decoding. VAS is typically measured as the ability to report back briefly presented strings of five letters (e.g., R H S D M) and has been shown to be related to reading performance in children both with and without dyslexia in various languages [29–33]. For adults, little data is available on VAS, although fMRI studies indicate that visual attention does play a role in adults both in reading in general and in dyslexia [34,35]. We might expect normal readers to outperform students with dyslexia on this task. Malingerers might have difficulty associating this task with dyslexia, and might thus resemble typical readers, or they could think that students with dyslexia might have difficulty with naming all the letters because of the speed of presentation, and perform too poorly on the task. In addition to letter strings, we presented digit strings. Similar to the predictions for RAN, malingerers might be expected to simulate dyslexia on naming letter strings more than digit strings, resulting in a specific pattern of performance on the VAS task.

Finally, we administered a measure of verbal intelligence. These skills do not play a role in diagnosing dyslexia, although it has been shown that adults with reading disabilities obtain poorer scores than adults without reading difficulties [36]. Malingerers might be tempted to feign dyslexia also on this task if they associate broader verbal skills with dyslexia. However, similar to phonological awareness, verbal intelligence tasks are oral tasks, using only spoken language, so malingerers might also assume that there is no relation with reading and spelling performance.

Taken together, the main aim of the current study was to compare performance on literacy and related cognitive skills for three groups: students with dyslexia, and typical readers, half of whom were asked to simulate dyslexia during testing. In addition, we tested how well malingerers could be distinguished from both students with dyslexia and typical readers using a limited number of tasks on which performance was found to differ across groups. The study was presented to both students and test administrators as a study of reading performance of students in higher education. Test administrators were thus unaware of the group assignment of the participants, as well as the true aim of the study. When participants subscribed to the study, a research coordinator randomly assigned students without dyslexia to the malingering and control groups, keeping an equal division of age and gender. Students in the simulation condition were emailed to ask them to malinger dyslexia during testing and to prepare themselves for testing by gathering information about dyslexia. The study thus differs slightly from previous studies in which malingerers either had no preparation [8] or were presented with information and instructions on dyslexia and its symptoms [4–6]. Asking students to find their own information on dyslexia and decide on their strategies before testing more clearly reflects what actual malingerers might do before testing. Based on previous studies, it was hypothesized that malingerers would be able to feign the core symptoms of dyslexia, and thus to fulfil the criteria for a diagnosis of dyslexia, although they might exaggerate the symptoms [4–6]. It is expected that they would have more difficulty simulating dyslexia to the same extent on different versions of a task, as well as on underlying cognitive skills, since these skills are less clearly associated with dyslexia and therefore less likely to evoke malingering strategies [6]. As a result, we expected to be able to distinguish malingerers from students with dyslexia and typical readers with adequate sensitivity and specificity.

## Method

### Participants

#### Recruitment and group assignment

Participants were recruited through flyers announcing a study of the literacy skills of students in higher education. Flyers were distributed around campus and student counselors were asked to display the flyer at their office to reach students with dyslexia. Volunteers were asked to contact the research coordinator, a trained research assistant who supervised the project together with the first author. The project coordinator then emailed the participant a background questionnaire. Based on the background information, the project coordinator assigned the participant to one of the groups and to a timeslot. Students self-reporting as having a diagnosis of dyslexia comprised the ‘students with dyslexia’ group. Students without a diagnosis of dyslexia were divided into subgroups by gender, age, undergraduate status, and study specialization. Subsequently the typical readers within each subgroup were randomly assigned to either the ‘typical readers’ or the experimental ‘malingerers’ condition, thus keeping a similar division in age, gender and year and specialization of their studies. Students assigned to the experimental malingering condition received an additional email asking them to simulate dyslexia and providing them with some suggestions on how to prepare for testing. In all, the initial sample included 81 students, with 19 in the Students with dyslexia group, 30 in the Malingerers group, and 32 in the Typical readers (control) group.

#### Verification of group assignment

Based on the scores on word reading, pseudoword reading and spelling, we checked whether participants with dyslexia actually did fit the diagnostic criteria for dyslexia and whether participants without dyslexia did not. We applied the criteria often used in the Netherlands, that is either a score among the lowest 10% in reading (words and/or pseudowords), or a score among the lowest 16% in reading combined with a score in the lowest 10% on spelling [37]. Three of the students with dyslexia (15.8%) did not meet these criteria for dyslexia, whereas four participants from the control group did (12.5%). For the students with dyslexia this might indicate that they feigned symptoms during their diagnostic assessment, or that they received treatment for their symptoms and improved their literacy skills. Both are reasons not to include them in the analyses as they do not currently show reading and/or spelling difficulties. Poor performance in the control group could indicate that these participants have undiagnosed dyslexia, or could indicate suboptimal performance (see for example [38]), both of which were reasons to exclude them from the control group of students with average to good literacy skills. For the malingerers, we established their actual reading level with an additional word reading test, but we did not have a measure of their actual spelling performance. Based on the actual word reading performance, one of the malingerers fitted the criteria for dyslexia. Furthermore, one of the malingerers reported that he was not able to simulate dyslexia, because he did not have time to prepare himself before testing. Data of both malingerers were excluded. In sum, data from all these participants (nine in total) was left out of the analyses.

#### Final sample characteristics

After verification, our final sample included 72 participants: 16 in the Students with dyslexia group, 28 in the Malingerers group, and 28 in the Typical readers (control) group. The characteristics of these participants are presented in Table 1. All participants were undergraduate or graduate students at an institute for higher education, either a university or a college of applied sciences. All students were fluent in Dutch, although nine students were raised bilingually, and three students reported English rather than Dutch as their preferred language. Most students were native speakers of Dutch and reported Dutch as their dominant language. The groups did not differ in age, *F*(2,69) = 0.95, *p* = .430, gender composition, χ²(2, *N* = 72) = 1.51, *p* = .471, percentage of university students, χ²(2, *N* = 72) = 0.03, *p* = .984, percentage of undergraduate students, χ²(2, *N* = 72) = 1.84, *p* = .398, or percentage of bilinguals, χ²(2, *N* = 72) = 1.47, *p* = .480. The groups did differ in reading ability, *F*(2,69) = 28.25, *p* < .001. As expected, students with dyslexia obtained the lowest word reading scores. However, contrary to expectations, malingerers, who are actually typical readers, also scored significantly worse than typical readers.

**Table 1:** Characteristics of the participants.

	Malingerers (*n* = 28)	Students with dyslexia (*n* = 16)	Typical readers (*n* = 28)
Age (*SD*)^1^	22; 7 (18.74)	22; 5 (25.21)	23; 1 (24.11)
% Male	28.6	12.5	25.0
% University	85.7	87.5	85.7
% Undergraduate student	82.1	87.5	71.4
% Bilingual	17.9	12.5	7.1
Word reading ability^2^ (*SD*)	103.36 (11.89)	85.94 (11.22)	112.29 (10.43)

^1^ Mean age in years; months and standard deviation in months.

^2^ number of words read correctly

### Materials

Before testing, a questionnaire was emailed to the participants to obtain some background information. During testing, a total of 11 tasks were administered; five tasks were used to examine literacy skills, and six tasks for underlying cognitive skills. After testing, malingerers were asked to fill out a debriefing form. The tasks are discussed in that order.

#### Background questionnaire

A questionnaire was emailed to the participants including a number of background questions, such as date of birth, gender, native language, year and specialization of their studies, as well as availability for testing. Students were also asked to indicate whether they had an official diagnosis of dyslexia. The answers were used to assign students to the groups, and to match the groups of malingerers and typical readers as closely as possible.

#### Literacy skills

**Word reading fluency**: Word reading fluency was assessed with the One Minute Test [39]. This test is often used as a measure of decoding abilities, both in schools and in diagnosing dyslexia. Participants were asked to read aloud a list of 116 words of increasing difficulty. They were asked to read as quickly and accurately as possible for 1 minute. The score consisted of the number of words read correctly. Percentile scores were calculated using students at the highest level of secondary education in the Netherlands (i.e., VWO) as the reference group, as these were the most fitting norms available [40]. After the test session, during debriefing (see Procedure), the parallel version of this task was administered to all participants, mainly to assess the actual reading fluency of the malingerers. Test-retest reliability is between .89 and .92 [39].

**Pseudoword reading fluency**: Pseudoword reading fluency was assessed with the Klepel [41]. This test is also often used as a measure of decoding ability in schools and in diagnosing dyslexia. Participants were asked to read aloud a list of 116 pseudowords of increasing difficulty. They were asked to read as quickly and accurately as possible for 2 minutes. The score consisted of the number of items read correctly. Similar to word reading fluency, percentile scores were calculated using the norms of Kuijpers et al. [40]. Test-retest reliability is .91 [41].

**Spelling**: Spelling accuracy was assessed with a spelling to dictation task of the Gl&schr [42]. The Gl&schr is a test battery designed to diagnose dyslexia among students in secondary school or higher education. The experimenter read aloud words that students were asked to write down as accurately as possible, without any time constraints. A total of 40 words was dictated, as well as 10 pseudowords. The score consisted of the number of items written correctly. Percentile scores were calculated using the norms available for students in higher education [42]. Split-half reliability is between .69 and .80 [42].

**Word reading accuracy**: A word reading accuracy task was used as an additional task for reading performance. Participants were asked to read aloud words that were presented on a computer screen for 100 ms. The task consisted of 39 words and 10 practice items selected from the One Minute Test [39]. The items were one to four syllables long. The score consisted of the number of words read correctly. This task was lent to us by a colleague who works in an institute specialized in diagnosing dyslexia, because it is sometimes used there in diagnosing dyslexia. Both average and poor readers have been shown to be able to identify these items accurately in 100 ms and thus to perform near ceiling on this task. The task could therefore function as a performance validity test. If indeed students with dyslexia and typical readers perform near ceiling, but malingerers do not, the task could help to determine whether the performance of the malingerers reflects their actual level of ability.

**Text reading time**: Text reading speed was included as an additional task for reading performance. Participants were presented with a short text (404 words), that was designed for the purpose of this study based on a text reading task by Gagliano et al. [43]. Participants were asked to read the text silently. To ensure that the text was read carefully, six small tasks (e.g., clap your hands) were included in the text, and a control question was asked when participants had finished reading. The score consisted of the time needed to read the text (in seconds), which was marked by the execution of the final task in the text. Text reading speed, especially when reading silently, is not usually examined in diagnosing dyslexia. However, the study by Gagliano et al. [43] indicated that text reading speed is especially important when diagnosing dyslexia among adults.

#### Underlying cognitive skills

**Phonological awareness**: Phonological awareness was assessed with a phoneme deletion task [44]. Participants heard a pseudoword, which they were asked to repeat. Next, the pseudoword was presented again, and participants were asked to delete one phoneme (e.g., ‘fral’ without ‘f’). They were presented with a total of 12 items. The first four items were monosyllabic pseudowords, the next four items were bisyllabic pseudowords, and the final four items were bisyllabic pseudowords with the phoneme to be deleted included twice (e.g., ‘stisnalt’ without ‘t’). Three practice items preceded the first eight items, and another two were presented before the final four items. The task was programmed in E-prime 2.0 [45]. The experimenter registered the response time and accuracy for each item by pressing the space bar and ‘+’ or ‘-‘ respectively. The score consisted of the number of items correct per minute.

**RAN**: RAN was assessed for letters (a, d, o, p, s) and digits (2, 4, 5, 8, 9) using the Test of Continuous Naming and Word Reading [46]. Participants were presented with a sheet of five columns with ten items each. They were asked to name all items as quickly and accurately as possible. The score consisted of the number of items named correctly per second. Split-half reliability is .87 for digits and .82 for letters [46].

**Stroop**: The Stroop task [47] included three conditions. For each condition participants were presented with a sheet with ten rows of ten items each, which they were asked to name as quickly and accurately as possible. First, participants were presented with color names (i.e., Dutch words for green, yellow, red and blue) printed in an incongruent color ink. They were asked to read the words (‘Stroop words’). From the same sheet, participants were then asked to name the colors of the ink (‘Stroop colors of words’). Finally participants were presented with colored boxes and were asked to name the colors (‘Stroop colors’). The scores consisted of the number of items named correctly per second. We also calculated the interference effect, by subtracting Stroop colors from Stroop colors of words.

**VAS**: VAS was assessed with the whole report task as designed by Valdois and colleagues [32]. Participants were first presented with 10 five-letter strings (e.g., B R T P S) and then with 10 five-digit strings (e.g., 8 4 2 1 9). They were asked to repeat the strings as accurately as possible, in the correct order. The strings were created from ten consonants and ten digits, all presented once in each position. The task was presented using Microsoft PowerPoint 2010. First, a plus sign was presented for 1000 ms to focus attention. Then, a string was presented for 200 ms in 28 point Arial font. The scores consisted of the number of letters and digits repeated correctly, with a maximum of 50 each.

**Verbal reasoning**: Verbal reasoning was assessed with the Similarities subtest from the Wechsler Adult Intelligence Scale III-NL [48]. Participants were presented with two words or concepts and were asked to describe how they are similar. After two practice items, a total of 14 items can be administered, although testing is discontinued after four consecutive incorrect answers. Each correct answer can be awarded one or two points. An additional 5 points are awarded for the first five items, which are only administered when the answers to the first two items are incorrect. The score consisted of the total number of points obtained, with a maximum of 33 points. Split-half reliability for this subtest is .84 [48].

**Verbal memory**: Verbal memory was assessed with the Digit Span subtest, also from the WAIS III-NL [48]: according to the manual, this digit span test is a measure of verbal memory. For the first part of the task, the experimenter read aloud sequences of digits, that participants were asked to repeat in the same order. For the second part of the task, participants were asked to repeat the digits in reversed order. The task thus captures verbal short-term memory and working memory, both in the auditory modality. Sequences increased from two to nine digits for digit span forward and from two to eight digits for digit span backward, with two sequences of each length. The tasks were discontinued when both sequences of a certain length were repeated incorrectly. The score consisted of the total number of sequences repeated correctly. Split-half reliability is .91 [48].

#### Debriefing

After testing, malingerers were asked to fill out a debriefing form. They were asked whether they had prepared themselves for testing, and if so, how they had prepared and for how long. They were also asked what their overall strategies were in simulating dyslexia, and whether they thought they had succeeded in simulating dyslexia. Next, they indicated for each task in the test battery whether they had feigned dyslexia on that particular task, and if so, how.

### Procedure

The tasks were administered by two trained research assistants, who were unaware of the true aim of the study, as well as the group assignment of the participants. Testing took place in a quiet room at the university. The test session lasted about 1 hour. Afterwards, participants met with the research coordinator for debriefing. They received a gift certificate of ten euros for participation. To optimize effort in preparation as well as during testing, additional gift certificates of fifteen euros each were awarded to the five students who were most successful in simulating dyslexia. When the study was completed the first author determined whose literacy scores most closely resembled those of students with dyslexia. All students were administered an additional timed word reading task, used to test the actual reading proficiency of the students who simulated dyslexia (see ‘word reading fluency’). The students who simulated dyslexia also filled out the debriefing form on the strategies they used to feign dyslexia during testing. Approval by the ethics committee of the Faculty of Social and Behavioral sciences of University of Amsterdam was obtained (file number 2016-CDE-6630). All participants provided written informed consent.

### Manipulation check

Malingerers indicated that they spent an average of 44.57 minutes (*SD* = 42.91 minutes, range 10–180 minutes) preparing before testing. They used a number of ways to prepare themselves (i.e., malingerers could use more than one strategy), the most common of which were looking on websites (78.6%), talking to friends or family members who have or know about dyslexia (53.6%), and watching movies, episodes or clips about dyslexia (28.6%). Most malingerers reported that they simulated dyslexia by reading and/or writing more slowly or less fluently (71.4%), and making mistakes (78.6%), for example by confusing letters (e.g., b/d) or digits, or by not applying rules for Dutch spelling. Four malingerers (14.3%) reported another specific strategy, that was either ‘looking cross-eyed’, ‘being nervous’, ‘appearing to be dumb’, or ‘often asking for repetition’. Concerning the specific tasks, at least 85 percent of the malingerers reported that they feigned dyslexia on each of the literacy tasks. On the underlying cognitive skills at least 75 percent reported that they had feigned dyslexia, with the exception of RAN digits (50%), Stroop colors (25%), verbal reasoning (39.3%), and verbal memory (50%).

When asked whether they thought they had succeeded in simulating dyslexia, 39.3% said ‘yes’, 17.9% said ‘a little’, 21.4% said ‘no idea’ and another 21.4% said ‘no’. Malingerers were unsure whether they succeed in simulating dyslexia, because they experienced difficulty simulating dyslexia: 39.3% indicated that it took ‘a lot’ of effort to simulate dyslexia, whereas 50% choose ‘some’, 3.6% choose ‘neutral’, and 7.1% choose ‘very little’ to indicate the amount of difficulty they experienced. As for the reason why malingering was difficult participants reported that it was hard to know how much simulation was necessary (i.e., ‘not to overdo it’, ‘not to appear lazy’ or ‘not to seem unmotivated’), that they did not know what it is really like to have dyslexia, or that they had to appear not to know things they actually did know.

### Analyses

First, we compared the three groups (i.e., malingerers, students with dyslexia, typical readers) in their performance on all the literacy and underlying cognitive skills using ANOVA, followed by Bonferroni post-hoc group comparisons to specifically compare malingerers to students with dyslexia and students with dyslexia to typical readers. When two tasks were used to measure the same skill, however, we started with a repeated measures ANOVA to examine whether the pattern of performance on variations of the same task was the same for each group. Second, we conducted discriminant analysis to obtain an optimal classification of the three groups. Followed by an analysis of the sensitivity and specificity of each of the separate measures included in the discriminant analysis.

## Results

### Data cleaning

Before running analyses we checked the data for outliers (i.e., scores more than three standard deviations above or below the group mean). However, we did not remove outliers from the dataset, because we were interested in individual differences in performance, as well as group comparisons. It should be noted that the variation in scores was large, and that therefore very few scores could be considered an outlier. In the sections below we discuss the possible impact of outliers on the results when outliers were identified.

### Literacy skills

First, we compared the three groups on reading and spelling performance. We used three tasks as diagnostic criteria (i.e., word reading, pseudoword reading, spelling), and included two experimental reading tasks (i.e., word reading accuracy, text reading time). The means and standard deviations are reported in Table 2. For all five tasks we found a stepwise pattern, with malingerers obtaining the lowest scores, followed by students with dyslexia, and typical readers obtaining the highest scores. In addition, for all tasks, the variation in scores was much higher among the malingerers than among either the students with dyslexia or the typical readers, indicating that performance varied greatly among malingerers.

**Table 2:** Performance on literacy tasks.

*Task*	Malingerers (*n* = 28)		Students with dyslexia (*n* = 16)		Typical readers (*n* = 28)		*η*_p_^2^	Malingerers vs. Students with dyslexia	Students with dyslexia vs. Typical readers
	M (*SD*)	Range	M (*SD*)	range	M (*SD*)	range			
Word reading^1^	49.86 (17.57)	15–83	82.06 (8.48)	67–98	105.32 (8.68)	90–116	.79	< .001	< .001
Pseudoword reading^1^	55.64 (21.79)	10–102	68.63 (9.88)	46–81	101.25 (8.62)	83–112	.65	.026	< .001
Spelling	28.46 (8.92)	6–42	35.00 (3.83)	28–41	40.14 (6.24)	13–46	.36	.013	.068
Word reading accuracy^1^	29.14 (9.85)	3–38	36.50 (1.59)	33–38	37.36 (1.13)	34–39	.29	.001	1.00
Text reading time	133.86 (60.46)	46–340	104.56 (23.75)	74–173	71.39 (13.05)	51–97	.33	.069	.032

All analysis of variance *F*-tests were significant at *p* < .001. Bonferroni-adjusted *p*-values are reported for the post-hoc comparisons.

^1^ number of items read correctly

The results of the ANOVA tests and post-hoc comparisons are presented in Table 2 as well. For word and pseudoword reading, all three groups indeed differed significantly from each other. The low scores of the malingerers were primarily due to low reading speed; they did not make more errors on the reading tasks than did students with dyslexia. Interestingly, malingerers obtained higher scores on pseudoword than on word reading fluency, whereas the opposite was true for typical readers and students with dyslexia. A repeated measures ANOVA indeed showed a significant interaction between group and type of reading task, *F*(2,69) = 16.49, *p* < .001, *η*_p_^2^ = .32. For spelling, students with dyslexia did not differ from typical readers, but malingerers scored significantly lower than both other groups. However, one of the typical readers scored particularly low on the spelling test. When that participant was left out of the analysis, students with dyslexia (*M* = 35.00) did score significantly lower than typical readers (*M* = 41.15, *p* < .01). The spelling score of one of the malingerers was exceptionally low and identified as an outlier. Removing this participant from the analysis did not change the results.

**Fig 1:**
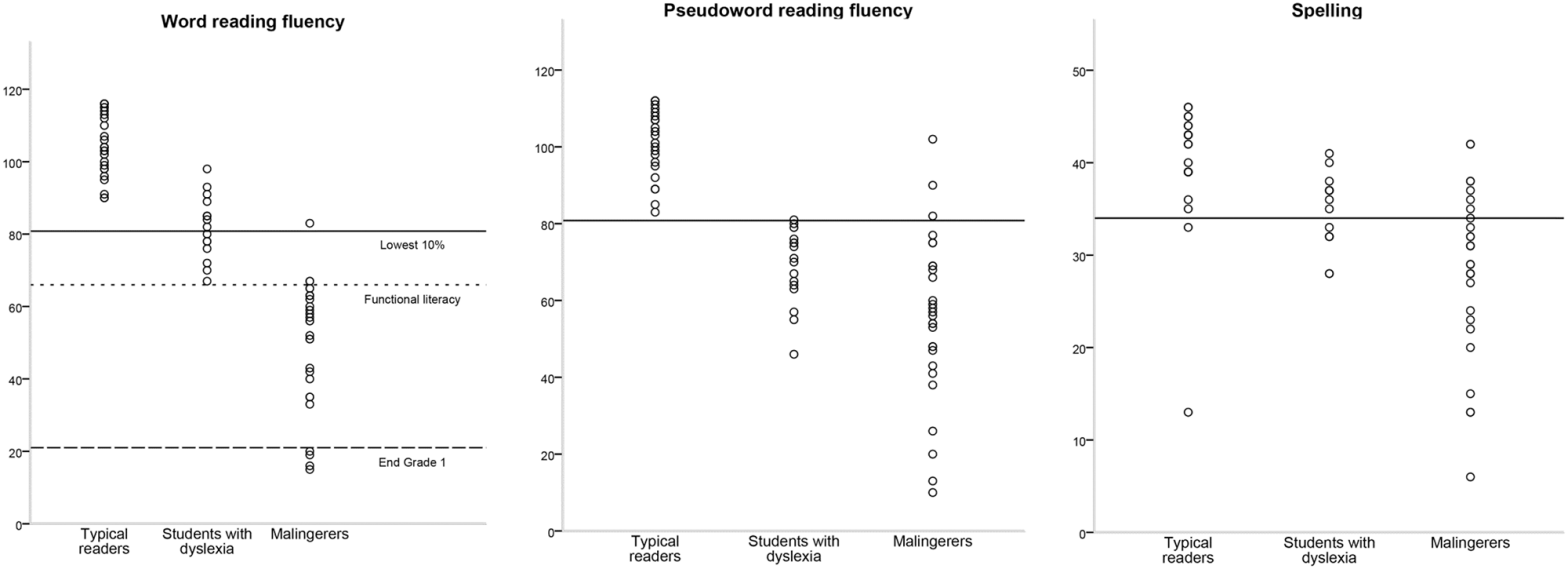
Scores on word reading fluency, pseudoword reading fluency and spelling for each participant. Scores below the bold line are within the lowest 10%. For word reading, scores below the dotted line fall below the level of functional literacy (Grade 4), and scores below the dashed line fall below the reading level normally obtained by the end of Grade 1.

In Fig 1 the scores on word reading fluency, pseudoword reading fluency and spelling are presented for each participant. Importantly, based on their scores on the diagnostic reading and spelling tasks, all but one of the malingerers (96.4%) would get the diagnosis of dyslexia. Those 27 malingerers all scored among the lowest 10% on at least one of the reading tasks, and 21 malingerers (75%) also scored among the lowest 10% on spelling. Interestingly, only 6 out of 16 students with dyslexia (37.5%) scored among the lowest 10% on spelling, indicating that most of them primarily presented with reading difficulties. The performance of the malingerers was notable in one important way; their word reading scores were exceptionally low. Twenty-five malingerers (89.3%) read fewer than 66 words, which is the average number of words read by children near the end of Grade 4 [39], and considered to be a functional literacy level. Four malingerers even read fewer than 21 words, which is the average number of words read by children by the end of Grade 1.

Two additional reading tasks were administered. These tasks are not used as regularly as the diagnostic tests. It was examined whether these tasks allow for a better distinction between students with dyslexia and malingerers. On text reading time, malingerers did not differ significantly from the students with dyslexia, but both groups read significantly more slowly than the typical readers. The reading time of one malingerer was exceptionally long and could be considered an outlier, but removing this score from the analysis did not change the results. Two malingerers read exceptionally slowly and two others read exceptionally fast, but overall, malingerers and students with dyslexia cannot be distinguished on the basis of text reading time. In contrast, on word reading accuracy, malingerers scored significantly lower than the students with dyslexia, who did not differ from the typical readers. The exceptionally low scores of three malingerers could be considered outliers on this task, but removing these scores from the analysis did not change the results. Scores on the word reading accuracy task appeared to be useful in distinguishing between students with dyslexia and malingerers. Whereas all the students with dyslexia and the typical readers read more than 80% of the words correctly, 13 malingerers (46.4%) read fewer than 80% of the words correctly. Four malingerers (14.3%) even read fewer than 50% of the words correctly.

### Underlying cognitive skills

Next, we compared the three groups on measures of cognitive skills that are related to literacy skills, including ‘usual suspects’ (i.e., phonological awareness, RAN, and verbal short-term memory), two ‘new kids on the block’ (i.e., Stroop and VAS), and verbal reasoning. The means and standard deviations are reported in Table 3. Similar to the results on reading and spelling, we found a stepwise pattern for all tasks, with malingerers obtaining the lowest scores, followed by students with dyslexia, and typical readers obtaining the highest scores. For most, but not all tasks, the variation in scores was again higher among the malingerers, than among either the students with dyslexia or the typical readers.

**Table 3:** Performance on underlying cognitive skills.

*Task*	Malingerers (*n* = 28)		Students with dyslexia (*n* = 16)		Typical readers (*n* = 28)		*η*_p_^2^	Malingerers vs. Students with dyslexia	Students with dyslexia vs. Typical readers
	M (*SD*)	Range	M (*SD*)	range	M (*SD*)	Range			
Phonological awareness	14.60 (6.67)	1.89–30.77	16.32 (5.52)	6.77–26.71	29.10 (8.34)	12.54–46.15	.49	1.00	< .001
RAN Letters	1.38 (0.60)	0.39–2.78	2.64 (0.54)	2.08–4.17	3.28 (0.60)	2.38–4.90	.69	< .001	.003
RAN Digits	1.57 (0.70)	0.46–2.63	2.50 (0.51)	1.92–3.57	3.24 (0.52)	2.17–4.55	.62	< .001	< .001
Stroop words	1.24 (0.48)	0.36–2.08	2.05 (0.23)	1.77–2.48	2.43 (0.29)	1.89–2.94	.69	< .001	.004
Stroop colors of words	0.95 (0.36)	0.38–1.61	1.14 (0.22)	0.81–1.47	1.51 (0.23)	0.99–2.00	.45	.134	< .001
Stroop colors	1.48 (0.38)	0.74–2.44	1.64 (0.27)	1.09–2.22	2.02 (0.36)	1.41–2.78	.33	.441	.003
VAS letters	36.50 (7.35)	20–50	37.81 (5.55)	25–48	45.04 (5.39)	32–50	.30	1.00	.001
VAS digits	41.39 (7.96)	17–50	42.81 (4.46)	35–50	47.07 (4.04)	36–50	.16	1.00	.077
Verbal reasoning	27.46 (3.04)	21–33	29.88 (2.16)	24–33	30.29 (2.03)	24–33	.22	.009	1.00
Verbal memory	13.75 (3.53)	7–21	13.31 (2.60)	10–20	18.14 (3.62)	10–24	.31	1.00	< .001

All analysis of variance *F*-tests were significant at *p* < .001. Bonferroni-adjusted *p*-values are reported for the post-hoc comparisons.

A repeated measures ANOVA including both RAN tasks showed that there was a significant interaction between group and RAN task, *F*(2,69) = 5.79, *p* = .005, *η*_p_^2^ = .14. Whereas both typical readers and students with dyslexia performed equally on the two RAN tasks or were slightly faster in naming letters, malingerers were slower on RAN letters than on RAN digits. Separate ANOVAs indicated that for both RAN letters and RAN digits malingerers scored significantly lower than students with dyslexia, who scored lower than the typical readers. Poor performance in malingerers was mainly due to slow naming speed, although three malingerers made four or more errors, which is exceptional, since both typical readers and students with dyslexia made zero errors or at most one error.

On phonological awareness, malingerers did not differ significantly from students with dyslexia, but typical readers outperformed both groups. Malingerers did make slightly more errors and answered slightly more slowly than students with dyslexia, but these differences were not significant. Separate analyses of accuracy and speed resulted in the same pattern of typical readers outperforming malingerers and students with dyslexia, who did not differ from each other.

A repeated measures ANOVA including all three Stroop tasks showed that there was a significant interaction between group and Stroop task, *F*(2,69) = 40.86, *p* < .001, *η*_p_^2^ = .54, caused by the relatively low scores of the malingerers on Stroop words compared to the other two tasks. On Stroop words, all three groups differed significantly from each other, with the malingerers naming the fewest items per second, followed by students with dyslexia, and typical readers obtaining the highest fluency. However, on naming the color, either in Stroop colors of words or Stroop colors, malingerers did not differ from the students with dyslexia, but students with dyslexia performed worse than the typical readers. Again, malingerers were mainly slow, but six malingerers made a striking number of errors (i.e., more than the maximum of three errors in typical readers and students with dyslexia), especially on Stroop colors of words. Surprisingly, groups did not differ significantly in the interference effect, *F*(2,69) = 0.10, *p* = .905, *η*_p_^2^ = .00. The interference effect was similar for malingerers (*M* = -.53, *SD* = .24) and students with dyslexia (*M* = -.51, *SD* = .16). Students with dyslexia also did not differ from typical readers (*M* = -.50, *SD* = .24).

A repeated measures ANOVA including both VAS tasks showed that there was no interaction between group and VAS task, *F*(2,69) = 2.22, *p* = .117, *η*_p_^2^ = .06. Separate ANOVA results indicated that on VAS letters, malingerers did not differ from students with dyslexia, but students with dyslexia named significantly fewer letters than the typical readers. On VAS digits, malingerers named fewer digits correctly than typical readers. The scores of the students with dyslexia did not differ significantly from the typical readers. However, the scores of two malingerers on VAS digits were exceptionally low and could be considered outliers. When these scores were removed from the analysis, the results were the same as for VAS letters; malingerers (*M* = 43.15) did not differ from students with dyslexia (*M* = 42.81, *p* > .05), but typical readers outperformed both groups.

Finally we assessed verbal reasoning and verbal memory. For both tasks, there were significant group differences. On verbal reasoning, malingerers scored significantly lower than students with dyslexia, who did not differ from the typical readers. However, as can be expected from participants in higher education, scores on this task were quite high, as all scores were above the 25^th^ percentile [48]. On verbal memory, in contrast, malingerers did not differ from students with dyslexia, but students with dyslexia scored significantly lower than the typical readers. On this task, several scores were at or below the 10^th^ percentile [48]. However, these scores were obtained in all three groups.

In conclusion, we found that malingerers performed more poorly on word and pseudoword reading than students with dyslexia. In addition, malingerers performed better on pseudoword reading than on word reading, whereas this difference tended to go in the opposite direction for students with dyslexia. Similar patterns were found for rapid naming. Malingerers were slower on rapid naming than students with dyslexia. They also named letters more slowly than digits, whereas naming letters and digits did not differ for students with dyslexia. Finally, malingerers performed remarkably poor on Stroop word reading, compared to their performance on the other two Stroop conditions. Differences on the other tasks were less clear.

### Classification

Since word and pseudoword reading and letter and digit RAN proved most useful in distinguishing between the groups, and these measures are commonly included in diagnostic assessment for dyslexia, we initially included these four measures in the discriminant analysis. The prior probabilities of all groups were set to equal. Two functions significantly differentiated the groups, λ = .151, χ²(8) = 127.78, *p* < .001, and λ = .747, χ²(3) = 19.72, *p* < .001 respectively. According to the structure matrix and group centroids, the first function represents all the tasks and distinguished typical readers (highest scores), from malingerers (lowest scores) and students with dyslexia (intermediate scores). The second function mainly represents pseudoword reading, which is particularly low in students with dyslexia compared to the other two groups.

With these functions, 93.1% of the participants were classified correctly. In the separate groups, classification was correct for 96.4% of the typical readers, 100% of the students with dyslexia and 85.7% of the malingerers. Of the malingerers, 10.7% were regarded as dyslexic and 3.6% as typical readers. Among the typical readers 3.6% were classified as dyslexic and none were classified as malingerers. In all, these results suggest that we can identify malingerers with reasonable sensitivity and specificity. The classification outcomes did not improve when we included only the diagnostic measures of dyslexia (i.e., reading and spelling), or the diagnostic measures plus the underlying cognitive skills.

We also examined the specificity and sensitivity of the separate measures. We first examined sensitivity using the lowest score obtained in the group of students with dyslexia as the cutoff score, thus ensuring 100% specificity. Sensitivity was high for word reading, with 89.3% of the malingerers identified as such. Similar sensitivity was found for RAN letters (89.3%). For RAN digits sensitivity was lower (67.9%). Pseudoword reading (25%), and spelling (32.1%) showed poor sensitivity. We cannot rule out that there were malingerers among the students with dyslexia. To allow for this possibility we also examined sensitivity at 90% specificity. This more lenient cutoff only slightly affected the results: word reading (96.4%), pseudoword reading (35.7%), spelling (42.9%), RAN letters (89.3%), RAN digits (67.9%). These findings indicated that apart from word reading and RAN letters, it is difficult to identify malingerers based on performance on a single task. However, sensitivity improves when performance across a number of diagnostic tasks is taken into account, and consistency in score patterns is considered in addition to separate scores. It should be borne in mind, however, that the current sample included only a small group of students with dyslexia that might not be representative. The specificity and sensitivity findings should therefore be interpreted with caution.

## Discussion

The availability of academic accommodations based on a diagnosis of dyslexia might evoke students to simulate the symptoms of dyslexia in the absence of true reading or spelling difficulties. In order to better understand potential malingering strategies, we examined whether students can simulate dyslexia. We assessed performance on literacy tasks and related cognitive skills in typical readers and in students instructed to malinger dyslexia, and compared them to students with dyslexia. We particularly searched for specific profiles and characteristics in performance on tasks commonly used in diagnostic evaluations of dyslexia in higher education.

First, we briefly zoom in on the students with dyslexia. Although only a small group of students with dyslexia was included in the study, the findings are in line with and add to a small body of research on students with dyslexia in higher education. In line with previous studies [49–51], students with dyslexia were found to differ from typical readers in reading, spelling, and phonological processing skills. Interestingly, pseudoword reading was found to be particularly important in distinguishing students with dyslexia from both typical readers and malingerers [5]. We also replicated the finding of Gagliano et al. [43] that students with dyslexia are impaired in silent text reading, in addition to the more commonly assessed oral (word) reading skills. Furthermore, students with dyslexia in higher education performed worse than typical readers on VAS, in line with previous studies indicating that visual processing skills do appear to play a role in adult reading performance [34,35]. However, it should be mentioned that it is still debated whether poor VAS performance is truly indicative of difficulties in visual processing skills, or whether phonological processes might also play a role, and performance on VAS rather reflects difficulties in establishing symbol-to-sound mapping [33,52]. Students with dyslexia showed deficits in verbal memory, similar to the findings by Swanson and Hsieh [36]. Counter to Swanson and Hsieh [36], the students with dyslexia did not differ from the control group on verbal reasoning.

Half of the students without reading or spelling difficulties were asked to simulate dyslexia during testing. Dyslexia is diagnosed when students show poor performance on reading and/or spelling tasks. When the criteria commonly used in the Netherlands were applied, the large majority of the malingerers would obtain a diagnosis of dyslexia. All but one of them scored within the lowest 10% on at least one of the two standardized reading tasks. Importantly, and in line with previous studies [4–6], malingerers exaggerated these core symptoms of dyslexia. They performed significantly poorer on literacy tasks than students with dyslexia. All but two of the malingerers even obtained a reading fluency level that is below the level of functional literacy. This level of performance is highly unlikely given that students attended higher education.

A similar pattern of students with dyslexia outperforming malingerers was found for spelling, letter and digit naming and word naming within the Stroop task. In contrast, malingerers were successful in simulating dyslexia on text reading, phonological awareness, VAS and verbal memory. Furthermore, about half of the malingerers performed extremely poorly on a word reading accuracy task. On this task, students with dyslexia did not differ from typical readers, indicating that this task might function as a performance validity test [4,5,7,8]. Poor performance on this particular task provides a contra-indication for dyslexia. Finally, some malingerers were found to make an exceptional number of errors on the naming tasks or to perform below average on verbal reasoning. In all, in line with findings of Harrison and colleagues [4,5], we found several indicators of feigning. It seems difficult to differentiate between students with dyslexia and malingerers based on a single test, but most malingerers can be identified with a combination of commonly used tasks.

Our expectations concerning the Stroop task were partly confirmed. We included a condition that is not commonly part of the Stroop task, as we asked participants to read the words on the card including words presented in an incongruent color ink. There were interesting group differences on this condition, in line with our findings on the literacy skills; malingerers were found to name these words particularly slowly, significantly slower than students with dyslexia. However, contrary to expectations, malingerers did not differ from students with dyslexia on the two standard versions of the Stroop task, naming the colors of words printed in an incongruent color, and naming colors separately, nor were differences found in the Stroop interference score, denoting the difference between these two conditions. In addition, the typical readers performed better on both tasks, but also did not differ in the interference score. Our results are thus not in line with the study of Protopapas et al. [25], who found that better reading skills are associated with less interference. This difference in findings might be due to the addition of the reading condition in our study, as the first Stroop task. Malingerers might have become aware of the potential role of reading fluency in the Stroop tasks, that usually evoke rather automatic responses. In hindsight it might have been better to administer the experimental version of the Stroop task after the standard versions.

Most importantly, malingerers displayed a particular pattern of results on several tasks, a pattern that differed from both other groups. Both typical readers and students with dyslexia were better at reading words than reading pseudowords and named letters and digits about equally fast. Malingerers, in contrast, performed better on pseudoword than on word reading, and better on digit than on letter naming. These four tasks are commonly used in diagnostic evaluations for dyslexia. Based on these tasks, 85.7% of the malingerers could be identified. In addition, no typical readers or students with dyslexia were incorrectly identified as a malingerer. These criteria thus seem to provide a reliable indication of whether an individual might be feigning dyslexia. The outcome could motivate the diagnostician to look more carefully into the specific difficulties of the client. We also examined the sensitivity and specificity of each task individually in identifying malingerers. These findings should be interpreted with caution, as our small sample of students with dyslexia might not reflect all potential score patterns obtained by students with dyslexia in particular, and adults with dyslexia in general. Nevertheless, the findings clearly indicated that performance on word reading and RAN letters can by itself identify about 90% of the malingerers correctly. The other tasks, however, were found to be far less sensitive, indicating that malingerers resort to a variety of strategies when feigning dyslexia. Therefore, it seems most promising to not consider separate scores, but to evaluate the consistency of performance across tasks to identify malingerers (see also [53,54]).

Although in the current study all students with dyslexia were correctly identified as such, a potential risk of using these types of analyses when diagnosing dyslexia is that students who actually do have dyslexia are incorrectly identified as malingerers. It is therefore important that an indication of malingering is either confirmed or falsified for instance in a conversation with the student in which he or she is confronted with the outcomes, or by closely examining a students’ history of dyslexia. In addition, further research is needed, with a large and representative sample of students with dyslexia to identify clear cutoff scores on these tasks that can be used in clinical practice. With the current study we have shown that it is possible to identify malingerers based on a small number of commonly used test, but our data was not sufficient to reach a combination of cutoff scores that can be reliably used in diagnosing dyslexia without a risk of misidentifying students who really do have dyslexia as malingerers.

The current study adds to previous findings on simulating dyslexia by encouraging a more authentic form of malingering and including an extensive test battery. However, there are also some limitations to this study. Most importantly, apart from reading performance we have no indication of the true performance of the malingerers (e.g., spelling, underlying cognitive skills). The malingerers were found to score more poorly than typical readers on reading ability, so it might be the case that their performance on various measures was not equal to that of the typical readers. We therefore did not compare these two groups directly. However, the performance of the malingerers on most tasks was exceptionally low and therefore still much lower than expected based on their ‘true’ reading ability. Nevertheless, in future studies it would be helpful to test malingerers both in a feigning and in a control condition. The difference in true reading ability between the malingerers and the typical readers in our study was much larger than expected, since they were randomly assigned to the conditions. This could indicate that the true reading ability of the malingerers, tested immediately after the test session in which they simulated dyslexia, might have been underestimated. Students might have had some trouble letting go of their malingering strategies and returning to their true level of performance. Therefore, it might be important to administer two test sessions, but on separate days and in a counterbalanced order.

Related to that, we did not have an indication of the effort the participants put into testing. As it has been shown that students who volunteer for research might not perform at an optimal level, and suboptimal effort is an important source of variance in performance, especially in college student populations, we would consider including a test of effort in future studies [38,55]. We did ask the malingerers to indicate how much time they spent preparing before testing. We used this information to get a first idea of how effort might have affected the findings. We divided the group of malingerers into groups who either spent ‘little’, ‘average’ or ‘much’ time preparing and compared these three groups to the students with dyslexia. We found a very clear pattern. All three groups of malingerers tended to either differ or not differ from the students with dyslexia, in line with the results reported. However, the malingerers who spent little time preparing actually performed more similarly to the students with dyslexia than those who spent more time preparing. Longer preparation thus seemed to be associated with more exaggeration in performance. These findings should obviously be considered carefully, but they further highlight the importance of manipulating or examining effort in future studies.

The current findings indicate that diagnosticians should look beyond test scores, norms and criteria, to try and obtain a complete picture of the client, his or her performance, and his or her position within society when diagnosing dyslexia. It seems important to obtain information about the history of the client, in terms of previous difficulties and attempts to treat these difficulties. This aspect of diagnosing dyslexia was not taken into account in the current study. However, it is an important criterion in diagnosing dyslexia that difficulties with reading and/or spelling originate in childhood [56], even though difficulties might increase in severity to the point where dyslexia is diagnosed in adolescence or even adulthood. Students attempting to simulate dyslexia might struggle to provide clear information and proof of early reading and spelling difficulties or early attempts to treat these difficulties, which could further substantiate suspicions of malingering.

The current findings increase our understanding of potential malingering practices among students in higher education, but it should be emphasized that there are no reliable estimates of exactly how often students simulate dyslexia to obtain a diagnosis and get access to academic accommodations. It is clear, however, that students seem quite able to simulate dyslexia when assessments for dyslexia are mainly confirmatory; students malingering dyslexia fulfill most criteria for dyslexia. However, their extremely poor performance, mainly on reading fluency and naming tasks, in combination with a deviant pattern in the scores on these tasks, are exceptional and should raise suspicion.

## Supporting information

S1 Data (SAV)
